# Are we ready for volume targeting during high-frequency oscillatory ventilation in neonates?

**DOI:** 10.1038/s41390-025-04015-y

**Published:** 2025-03-17

**Authors:** David G. Tingay, Sophia I. Dahm, Arun Sett

**Affiliations:** 1 Neonatal Research, Murdoch Children’s Research Institute, Parkville, VIC Australia; 2 Department of Paediatrics, University of Melbourne, Melbourne, VIC Australia; 3 Department of Critical Care, University of Melbourne, Melbourne, VIC Australia; 4 Department of Obstetrics, Gynaecology and Newborn Health, The University of Melbourne, Parkville, VIC Australia; 5 Newborn Services, Joan Kirner Women’s and Children’s, Sunshine Hospital, Western Health, St Albans, VIC Australia

High-frequency oscillatory ventilation (HFOV) is a mode of invasive respiratory support that delivers an oscillatory wave at a fast rate (5–20 Hz in neonates) around a constant distending or mean airway pressure (P_AW_), to achieve gas exchange with tidal volumes less than anatomical deadspace.^1–4^ HFOV is extremely effective when applied to the right lung conditions, especially severe acute respiratory failure related to atelectasis (such as surfactant-deficient respiratory distress syndrome) and pulmonary hypertension.^2,5–7^ As HFOV delivers small tidal volumes it has been advocated as a mode that may reduce volutrauma in severe lung disease.^2^ Consequently, HFOV is often used as a rescue treatment when conventional modes of ventilation (CMV) are not facilitating effective gas exchange or doing so with high pressures or volumes.^8^ The differences in setting ventilator parameters compared to CMV (due to different mechanism of action) have arguably made the effective application of HFOV at the bedside challenging.^2^

In this edition of *Pediatric Research*, Liu and colleagues undertook a systematic review to compare the efficacy and safety of HFOV with a volume-targeting mode (commonly termed ‘volume guarantee’; VG) and HFOV without VG.^9^ The authors identified 11 studies that met the inclusion criteria, of which only 3 were small randomised controlled trials (the remainder being one non-randomised controlled trial and 7 observational studies). Of the total of 537 neonates included the primary outcome of survival-free of severe bronchopulmonary dysplasia (BPD) if born <32 weeks’ GA was reported as an Odds Ratio (OR) of 3.15 (95% CI 1.66, 5.98) favouring HFOV + VG. Liu and colleagues also reported statistically significant benefits of HFOV + VG with regards to any BPD, duration of ventilation, length of hospital stay and common neonatal morbidities (such as air leak and intraventricular haemorrhage).

Due to the lack of large randomised clinical trials, the authors elected to include randomised and observational studies in their analysis. Most of the included studies primarily aimed to determine the effectiveness of HFOV + VG in either delivering the set V_T_ or stabilising CO_2_ or oxygenation rather than survival free of BPD or safety. The populations in the three small (*n* = 40–58 per study) randomised trials differed; 2 including term neonates with acute respiratory failure (one pulmonary hypertension and the other following cardiac surgery) and the other preterm infants. Like the population that receives HFOV generally, this creates a significant heterogeneity that must be factored when considering the outcomes.^8^ The observational studies compared historical control groups after practice change to HFOV + VG, and often with different durations of recruitment in each arm. This creates the risk that any benefit was related to other improvements in care rather than the intervention in question. Despite these limitations in quality of studies, the authors applied a structured and rigorous approach to their search strategy and analysis.

There is a clear need for systematic evaluation of HFOV + VG. Like volume-targeted ventilation during CMV, the premise of HFOV + VG is that the ventilator can rapidly adapt the pressure amplitude (ΔP) to maintain a constant tidal volume (V_T_), and thus stabilise carbon dioxide (CO_2_), better than a human. This is especially relevant during HFOV due to the real risk of rapid and large changes in CO_2_.^2,7^ Paradoxically, large CO_2_ changes could increase the risk of adverse outcomes such as intraventricular haemorrhage, air leak and lung injury. As a result, nearly all high-frequency oscillators now offer HFOV + VG, but this has occurred without prior scientific evidence. There are two important reasons why volume-targeting during HFOV cannot simply be extrapolated from the large body of evidence and experience using CMV + VG.^10^ Firstly, V_T_ is achieved at much lower values, usually between 1.5 and 3.5 mL/kg.^7^ Whether modern devices can accurately monitor V_T_ at <2 mL is unclear.^11^ Secondly, CO_2_ removal during HFOV is determined by the product of frequency and V_T_^2^, with V_T_ being dependent on both ΔP and frequency (during CMV, frequency and V_T_ are independent).^3,4^ Practically this means that the appropriate V_T_ for CO_2_ removal will be different as frequency changes. Encouragingly, HFOV + VG appears to achieve its algorithmic intentions. Although, V_T_ was only reported in 3 studies, one showed that V_T_ was more likely to remain in target during HFOV + VG compared to HFOV alone, another found a small reduction in V_T_ (approximately 0.2 ml/kg) and the last reported no difference in V_T_ levels. More clinically relevant was that hypo- or hypercapnia were less likely with HFOV + VG in the 5 studies that measured CO_2_, providing a clinical and physiological rationale for use.

Liu and colleagues have focused their review on trials of HFOV + VG versus HFOV without VG. This is a strength. Volume-targeting is not the mechanism of gas exchange during HFOV but simply an engineering algorithm to allow ΔP adaptation to maintain a constant V_T_. Thus, the impact of VG on any difference between HFOV and CMV with regards to mortality and important morbidities is likely to be incremental rather than substantive. It is also unlikely clinicians will choose to use HFOV only if it can be delivered with volume-targeting, especially as rescue treatment. This then leads to the question as to what the best outcome is for any comparison of HFOV with and without VG? The authors have used survival without severe BPD as the primary outcome. This is an admirable choice, and clearly relevant to clinicians. But the expectation that improving survival without severe BPD from stabilising CO_2_ via just one of multiple mechanisms of HFOV is optimistic. Especially as none of the included studies were designed to test this or involved term infants, and the statistically significant OR was calculated from only 2 prospective observational studies (*n* = 145). A more reasonable outcome may be one that speaks directly to the mode of action, such as duration of ventilation (as ΔP is a factor used to define extubation readiness), or safety (as the modality is ‘new’). HFOV + VG reduced the duration of invasive ventilation compared to HFOV (2 randomised trials and 3 observational studies; n = 256 infants); OR −1.47 (−2.00, −0.95). Similarly, HFOV + VG was also not associated with an increase in airleak.

There is a clear need for systematic evaluation of HFOV + VG, however the strength of a systematic review lies in the robustness of the included studies. In this case, the limitation of this systematic review is in the lack of available large randomised controlled trials and interpretation of statistical significance. The underlying body of work includes studies of small population numbers, varying study designs and poor quality. The risk of interpretation bias is thus high even when statistical tools are applied to account for heterogeneity. This highlights the need for the reader to always evaluate the study details, particularly when observational and randomised trials are combined, and apply caution regarding clinical generalisation, especially when confidence intervals are wide.

**Fig. 1:**
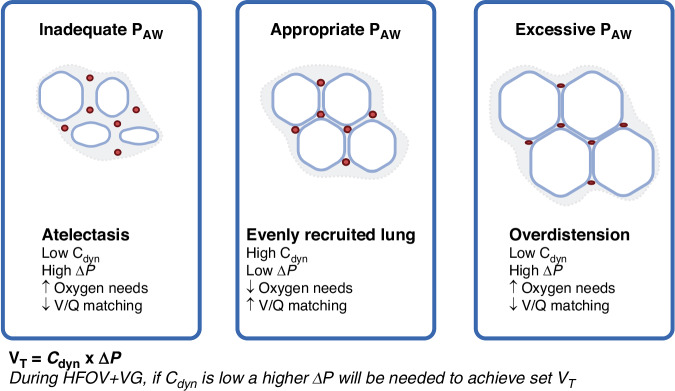
The importance of mean airway pressure (P_AW_) during HFOV with volume guarantee (VG). During HFOV, lung volume state is determined by P_AW_, which in turn influences lung compliance (C_dyn_), oxygenation and perfusion. Even if the set V_T_ is correct, inappropriate P_AW_ will result in unnecessarily high ΔP and uneven delivery of V_T_ during HFOV + VG. Tingay, D. (2025) https://BioRender.com/d56r333.

Like all modes of respiratory support in critical care, rather than simply using VG during HFOV it is how it is used that matters the most. It is possible to deliver any mode of ventilation in a harmful or optimal manner. For example, CMV + VG reduces BPD in preterm infants compared to CMV without VG in meta-analysis of large trials,^10^ but clearly setting an excessive V_T_ target would still be injurious irrespective of how V_T_ is controlled. This is no more evident in neonatology than the clinical trials of first-intention HFOV without VG to treat acute preterm lung disease compared to CMV.^12^ Meta-analysis has shown that not only must HFOV be applied with a ‘high-lung volume strategy’ but also that the benefits of either HFOV or CMV are greatest when that mode is applied ‘optimally’ and the other not.^13^ Unfortunately, the ventilator settings, especially P_AW_ values, use of a high-lung volume strategy, frequency, oxygen and CO_2_ targets, are not reported. This is a flaw that is common to HFOV meta-analysis.^14^ How then can a clinician ‘optimally’ set HFOV + VG? Physiologically rational starting V_T_ parameters are now available which emphasises two key points.^2^ Firstly, these are starting values and frequent re-evaluation based on CO_2_ clearance is essential. Secondly, as illustrated in Fig. 1, HFOV + VG without first correctly setting P_AW_ and frequency will negate any potential lung protective benefit.^2^

The availability and enthusiasm for HFOV + VG means the opportunity to systematically evaluate this mode in large clinical trials before wide-spread clinical uptake has almost certainly passed. Loss of equipoise, however, does not preclude further study. This review suggests that some benefits of HFOV + VG may be present. While the results of many observational studies of BPD have not been replicated in randomised controlled trials, well-designed randomised trials of HFOV including VG, or cohort studies with appropriate adjustments made for confounders in the absence of randomisation, are still possible.^15^ Volume targeting should be included as part of an optimal HFOV lung protective package, if they target relevant and well-defined populations and conditions (such as the most preterm). Until such evidence is available however, clinicians should not necessarily avoid using HFOV + VG but understand how it works and exercise a healthy degree of caution when applying this relatively new ventilation strategy.
